# Prenatal and early postnatal exposure to a natural disaster and Attention-Deficit/Hyperactivity Disorder symptoms in Indian children

**DOI:** 10.1038/s41598-022-20609-6

**Published:** 2022-09-28

**Authors:** Tomasz Hanć, Aleksandra Gomula, Natalia Nowak-Szczepanska, Raja Chakraborty, Sławomir Kozieł

**Affiliations:** 1 Institute of Human Biology and Evolution, Faculty of Biology, Adam Mickiewicz University, ul. Uniwersytetu Poznańskiego 6, 61-614 Poznan, Poland; 2 Department of Anthropology, Hirszfeld Institute of Immunology and Experimental Therapy, Polish Academy of Sciences, Wrocław, Poland; 3 Department of Anthropology & Tribal Studies, Sidho-Kanho-Birsha University, Purulia, West Bengal India

**Keywords:** Risk factors, Psychiatric disorders, Environmental impact, Psychology and behaviour, Cognitive neuroscience, Human behaviour

## Abstract

The aim of this study was to assess the relation between early exposure to stressful events and symptoms of the Attention-Deficit/Hyperactivity Disorder (ADHD) in children, based on the outcomes from a natural experiment. It was hypothesized that children pre- and postnatally exposed to cyclone Aila have increased ADHD symptoms compared to the control group, and the effect depends on the timing of the exposure. Indian children (8–11 years) prenatally (N = 336) and early postnatally (N = 216) exposed to cyclone Aila were compared to a non-exposed control peer group (N = 285). ADHD symptoms were assessed using the Conner’s Teacher Rating Scale Revised. The main effect of exposure to the cyclone on the total ADHD symptoms’ score, ADHD index, Hyperactivity and Oppositional symptoms was significant and independent to covariates: age and sex of children, gestational age and birth weight, maternal stress during the year before the study and the socioeconomic status of a family. The timing of exposure and sex of the children were found to be a significant moderator of the relation between early exposure to the natural disaster and ADHD symptoms. The prenatal, but also early postnatal exposure to stressful experiences such as a natural disaster, may disturb the development of cognitive functions and behavioural control, thus increasing the risk of ADHD in children.

## Introduction

The Attention-Deficit/Hyperactivity Disorder (ADHD) is characterized by age-inappropriate levels of inattention, hyperactivity and impulsivity^1^. It affects human life by increasing significantly the risk of academic failures, conflicts with peers and family members^2^. Lower occupational level^3^, unemployment^4^, physical injures and early life mortality^5^ were also shown to be among the adverse life outcomes associated with ADHD. Thus, ADHD is considered a life-long disorder bearing a high cost for the individuals, as well as for society.

Family, twin and adoption studies showed that ADHD is of high heritability. Nevertheless, the evidence for genes as risk factors for ADHD does not exclude the role of environment in the etiology of the disorder^6^. Previous studies on environmental factors found, among others, that the exposure of mothers to stressful events during pregnancy^7–9^ and a high level of perceived prenatal maternal stress (PNMS)^8^ were associated with an increased risk of ADHD. Research has also shown that adverse experiences in early postnatal life are associated with an increased probability of an ADHD diagnosis in children^10–12^. However, the early stress-ADHD association is difficult to interpret for several reasons. Although pre-/postnatal stress is commonly understood as one of the environmental factors, it was suggested that stressful experiences may depend to a varying degree on genetically influenced behavioural tendencies. Thus, their effects on a child’s development could not always be conceived as the pure effects of environmental factors^9^. Another problem with the interpretation of the relation between ADHD and early stress is that the cause of prenatal stress may persist into postnatal life, disrupting any further development of behavioural control^8^. It is unclear to what extent environment may be understood purely as the source of stressful experience independent to genes, and if the increased risk of ADHD is related to prenatal or postanal stress specifically or if it is rather the result of the accumulation of pre- and postnatal exposure to a stressor? Thus, for a better understanding of the early stress-ADHD link, the research on the effects of adverse events that are considered to be gene independent, e.g.: natural disaster^9^, and inclusion of the control of timing of exposure to a stressor into the analysis may be particularly helpful.

The aim of the research was to assess the relation between pre- and early postnatal exposure to a gene independent stressful event and ADHD symptoms in children. The study was based on outcomes from a natural experiment. An effect of the severe cyclonic storm Aila, which affected the coasts of Bangladesh and India in 2009, on ADHD symptoms have been examined. We hypothesized that Indian children pre- and postnatally exposed to the natural disaster had significantly increased ADHD symptoms in the preadolescent period compared to the control group of children non-exposed, and the effect of the adverse event depends on the timing of exposure.

## Materials and methods

### Ethical statement

Ethical approval for the research was obtained from the Institutional Ethics Committee for Research on Human Subjects, West Bengal State University, West Bengal, India (approval no. WBSU/IEC/14/03, dated 13.11.2017). The research was conducted in accordance with the Helsinki Declaration and The Protection of Children from the Sexual Offences Act of India. The data were collected from the eligible children and their mothers. Only children whose parents or legal guardians provided an informed written consent were included in the study.

### *Aila*:* the severe cyclonic storm*

A tropical cyclone called *Aila* hit India and Bangladesh at a speed of 120–140 km per hour, between May 23 to May 26 2009, and devastated the coastal islands of the Sunderbans, the largest delta in the world. According to the India Meteorological Department, it was classified as a ‘severe cyclonic storm’^13^. It claimed 138 human- and uncountable cattle lives and human properties^14,15^. Scientific studies conducted so far to portray the aftermath of the cyclone have mainly focused on the ecological^13,16^ and economical effects^17^, livelihood and resilience^18^, on the post-disaster health hazards, such as an increased number of diarrhoea cases and cholera outbreak and on the psychological impacts on the adult population^19^. Unfortunately, there is an acute lack of scientific evidence by systematic studies on the aftermath recovery from the cyclone Aila disaster in India. From the newspapers and other media reports, it is known that after the storm and the resulting inundation, the inhabitants had to stay in the temporary rescue camps on the adjacent Islands as well as on the mainland. Many people temporarily migrated to their relatives in safe unaffected areas of the state, if possible. Salt affected flood water remained on the Island for about the next 6 months^20^. All of the fishing areas were flooded and could not be restored for several months, until the water became normal again. Agricultural land were the worst affected as the salt from the flood water created a thin layer on the top soil which remained for about 5 years on average, until the fertility was regained gradually. Agricultural production has been greatly affected after cyclone Aila due to the high salinity and pH condition of the soil^17^.

### Study area and participants

The study included three groups of children (1) AilaPreS (prenatal exposure to cyclone Aila-related stress): recruited from the two islands of the Sunderban area most affected by the cyclone. These children were intrauterine during the cyclone and born between June 2009 and February 2010, (2) AilaPostS (postnatal exposure to cyclone Aila-related stress): the children who lived in the same areas as AilaPreS but were born up to 2 years before cyclone Aila and faced all the post-disaster hazards during their infancy, and (3) the control group belonged to the same birth cohort as AilaPreS, i.e., intrauterine during the cyclone and born between June 2009 and February 2010. They were recruited from the villages of the neighboring district that were not affected by the cyclone.

The AilaPreS and AilaPostS groups came from two islands of the Sunderban delta region, called Satjelia and Kumirmari, in the district of South 24 Parganas and under the community development (CD) block, called *Gosaba*. These were the most affected islands, in terms of the severity of the damage due to the cyclone. Children of the AilaPreS and AilaPostS groups were recruited from 22 schools on Satjelia Island and 13 schools on Kumirmari Island. The control group of participants were recruited from 21 schools located in the villages of the rural Eastern part of the adjacent district, North 24 Parganas, under the CD Block, Bongaon. Those villages were selected because they matched the island villages under the study in respect of ecology, occupation and the migration history of the population. The people of this area also highly resembled those of the areas affected by cyclone Aila with regard to the origin, culture and language^21,22^.

### Assessment of ADHD symptoms

The level of ADHD symptoms among the children was assessed by class teachers using the Conner’s Teacher Rating Scale Revised (short version) (CTRS-R:S)^23^. The Conner’s questionnaires belong to the most widely used child behaviour ratings scales in the world^24^, including India^25–27^. The symptoms were assessed using the total score of CTRS-R:S and 4 indexes: Cognitive problems/inattention index, Hyperactivity index, Oppositional symptoms index and ADHD index, which is a combination of items derived from empirical discrimination between clinical and matched control cases^23^. The sum of the scores for each index as well as for all items was used for the purpose of the statistical analysis.

### Confounding variables

Taking into consideration the possible high rate of illiterate parents of the children in the study, the structured interview method was applied to gather information on the basic characteristic of the sample and confounding variables. The questions/option statements were read to the mothers with adequate explanations in a standardized manner, and the research investigator filled in the forms or ticked the appropriate options.

The symptoms of ADHD vary between males and females in terms of severity and subtypes^28^. Thus, we assumed that sex could also act as a significant moderator of cyclone Aila exposure-ADHD symptoms link and so included this factor in the analysis as a confounding variable.

The level of maternal stress related to adverse life experiences which occurred during the year before the time of the study was assessed by the Holmes and Rahe Stress Scale (HRSS), also known as the Social Readjustment Rating Scale^29^. The questionnaire is a list of 43 stressful life events called ‘Life Change Units’. Each of them has a different ‘weighting’ for stress. The higher the number of stressful events and the larger the weighting of events, the higher is the level of stress.

ADHD has been previously found to be related to the low socioeconomic status (SES) of a family^30^ and, based on previous reports from the region^19^, we assumed that the economic situation of a family was acutely affected by the natural disaster. Previous studies showed that among inhabitants of the same area, the psychological trauma following cyclone Aila had differentially affected people from different socio-economic strata, the lower class being the worst affected^20^. Thus, SES was controlled in the statistical analyses of the present study. The monthly family income per capita for each family was calculated by dividing the monthly family income by the number of family members. The educational background of each parent was also recorded as the highest level which they passed from educational institutions and was categorised into three groups: non-literate, up to primary level, and up to secondary level, for the purpose of the analysis.

The risk of ADHD was found to be related to perinatal characteristics as the Apgar score, term of birth and birth weight in previous research^31^. Because gestational age and birth weight are associated with both prenatal stress and ADHD, they were included in the analysis as covariates.

### Statistics

Differences between the three groups were assessed by the one-way analysis of variance (ANOVA) in the case of a normally distributed dependent variable. However, for the dependent variables which were not normally distributed, the Kruskal–Wallis test was performed. Differences between sexes within each group were assessed by the student t-test for independent samples. Differences in the scores of ADHD and Oppositional symptoms between the exposed groups and control group within each sex were assessed by ANOVA, and *post-hoc* comparisons were done by the Dunnett’s test. Differences in distribution in the appropriate categories of parental education were assessed by Pearson’s chi-square test. The effects of exposure to cyclone Aila were assessed by a multiple analysis of covariance applied by the Generalized Linear Model (GLM) with the logit link function. Two GLM models of this analysis were performed, depending on the number of confounding factors that were included. In model I, scores of ADHD and Oppositional symptoms were dependent variables, while groups (AilaPreS, AilaPostS and control group) and sex were the independent variables and age was a covariate. In model II the following variables were included as the covariates: age, fathers’ and mothers’ education levels (in three categories each), mothers’ HRSS, family income per capita, gestational age at birth and birth weight. The second-order interaction effect between group and sex was additionally included in both models. The significance of the effects was assessed by Wald’s chi-square, and the odds ratios (OR) were reposted. The effect size was calculated using formula ln(OR)/1.81^32^. On all graphs, the results of the *post-hoc* comparisons between the groups were included. The required significance level was assumed at p < 0.05. All calculations were performed in Statistica 13.1.

## Results

A total of 987 children were initially identified and 927 of them participated in the study. Ninety children dropped out or were excluded from the study for different reasons, e.g.: lack of important information for the questionnaire or incorrect date of birth. The detailed description of the recruitment and selection procedure has been described elsewhere^22^. For the total number of 837 children information on the ADHD symptoms were available. Among them 336 children were included to the AilaPreS group, 216 children to the AilaPostS group and 285 children to the control group. However, due to the lack of other parental and birth information the numbers differed depending on the analysis and is reported in each table. The two main statistical GLM models I and II included 802 and 524 children, respectively. However, the children who dropped out from the main analysis did not significantly differ in most of the CTRS-R:S indices, except for the higher Cognitive problems/inattention symptoms (p < 0.05).

The analysis showed significant differences between AilaPreS, AilaPostS and control group in mean age, education of mothers, mean income *per capita,* scores of HRSS and gestational age (see Table 1).

**Table 1:** Descriptive statistics of all confounding variables by groups.

	AilaPreS	AilaPostS	Control	
**Age, N; mean (SD)**				
Boys	175; 8.06 (0.21)	109; 9.31 (0.50)	142; 8.31 (0.23)	F = 543.4***
Girls	161; 8.08 (0.24)	107; 9.28 (0.36)	143; 8.32 (0.23)	F = 647.9***
**Mother’s education, N (%)**				
Not educated	15 (5)	41 (23)	30 (11)	χ^2^ = 54.01***
At most primary	197 (67)	116 (65)	147 (55)	
At most secondary	83 (28)	21 (12)	91 (34)	
**Father’s education, N (%)**				
Not educated	48 (15)	27 (15)	42 (16)	χ^2^ = 4.60 n.s.
At most primary	168 (52)	101 (57)	125 (47)	
At most secondary	105 (33)	50 (28)	98 (37)	
**Income per capita (INR), N; mean (SD)**	300; 1207 (1090)	177; 844 (586)	263; 1666 (1203)	H = 130.77***
**Scores of last year mother’s stress (HRSS), N; mean (SD)**	295; 335 (136)	152; 117 (130)	228; 92 (84)	H = 344.41***
**Gestational age (weeks), N; mean (SD)**	311; 38.0 (2.3)	148; 37.6 (2.7)	257; 38.8 (2.7)	F = 12.45***
**Birth weight (g), N; mean (SD)**	261; 2662 (524)	150; 2678 (479)	239; 2749 (545)	F = 1.87 n.s.

*n.s.* non-significant, *AilaPreS* prenatally Aila-exposed children, *AilaPostS* postnatally Aila-exposed children, *Control* non-exposed to Aila counterparts, *H* result of the Kruskal–Wallis test, *F* F-statistic from ANOVA test, *χ*^*2*^ value of the Pearson’s’ chi square test.

***p < 0.001.

In the boys and girls, all CTRS-R:S indexes were significantly different between the groups (at least p < 0.01) with the exception of Cognitive problems/inattention in girls. The highest scores for all of the indices were found in the AilaPostS group and followed by AilaPreS. Total score, of the ADHD index and Hyperactivity index differed significantly between the boys and girls from the AilaPreS group (p < 0.001) to the disadvantage of the boys. Similarly, within AilaPostS and the control group, in all CTRS-R:S indices boys showed significantly higher scores than girls (at least p < 0.05), except for Cognitive problems/inattention (see Table 2).

**Table 2:** Descriptive statistics of the CTRS-R:S indices in the AilaPreS, AilaPostS and control group.

	AilaPreS			AilaPostS			Control group			ANOVA for differences between groups	
	N	Mean	SD	N	Mean	SD	N	Mean	SD	F	P
**Boys**											
CTRS-R:S total	170	35.1	16.8	101	37.9	14.9	137	28.9	17.9	9.28	0.001
ADHD index	173	14.3	6.8	105	15.3	6.0	139	11.6	7.4	10.17	0.001
Cognitive problems/inattention	171	7.1	4.4	107	7.7	3.6	141	6.0	3.8	5.93	0.01
Hyperactivity	173	8.8	4.6	108	9.3	4.1	142	7.3	5.2	6.84	0.001
Oppositional symptoms	172	4.5	3.3	107	5.5	3.1	142	3.9	3.9	7.09	0.001
**Girls**											
CTRS-R:S total	161	29.1^c^	14.6	98	30.3^b^	15.1	140	22.3^c^	17.3	9.90	0.001
ADHD index	161	11.7^c^	6.0	104	12.4^b^	6.1	140	8.9^c^	7.0	10.82	0.001
Cognitive problems/inattention	161	6.4	4.0	106	6.8	3.7	140	5.8	4.0	2.21	n.s.
Hyperactivity	161	6.4^c^	4.1	104	7.0^c^	3.7	140	4.6^c^	4.5	11.40	0.001
Oppositional symptoms	159	4.3	3.1	104	4.2^a^	3.4	141	2.7^b^	3.4	10.16	0.001

Differences were assessed by one-way ANOVA, and sex differences were assessed by the t-Student test for independent samples (marked at only significant p – level).

*AilaPreS* prenatally Aila-exposed children, *AilaPostS* postnatally Aila-exposed children, *Control* non-exposed to Aila counterparts, *F* F-statistic from ANOVA test, *p* the level of significance of result, a, b and c—intragroup statistically significant differences between girls and boys: ^a^p < 0.05, ^b^p < 0.01, ^c^p < 0.001.

Results of the analysis of covariance show the significant effect of group (AilaPreS, AilaPostS and the control group) on all CTRS-R:S indices (p < 0.05) in both of the analysed models. Moreover, all effects of second-order interactions between group and sex were not significant. The effect size assessed by OR (control group as a reference) indicated an increased risk of higher scores of all CTRS-R:S indices in the Aila-exposed groups in a range from OR = 1.03 to OR = 1.23 except for Cognitive problems/inattention score in the AilaPostS group in both models, where OR = 0.99, and Hyperactivity score in the AilaPreS group in model II, where OR = 0.99). The highest ORs were found for Hyperactivity index and AilaPostS in both model I and II (OR = 1.20 and OR = 1.23 respectively) (see Table 3).

**Table 3:** Results of the Generalised Linear Model (GLM), where CTRS-R:S indices were dependent variables, and groups (AilaPreS, AilaPostS, Control group) and sex were independent variables.

		Model I N = 802					Model II N = 524				
		Group	AilaPreS	AilaPostS	Sex	Group x Sex	Group	AilaPreS	AilaPostS	Sex	Group x Sex
CTRS-R:S total score	Wald’s χ^2^	28.83***			32.23***	0.61	12.56**			19.60***	0.88
	OR (± CI)		1.09 (1.01–1.17)	1.08 (0.98–1.19)				1.08 (0.96–1.21)	1.09 (0.95–1.25)		
	Size effect		0.05	0.04				0.04	0.05		
ADHD index	Wald’s χ^2^	31.12***			32.86***	0.41	15.28***			19.11***	0.68
	OR (± CI)		1.09 (1.01–1.18)	1.08 (0.98–1.19)				1.07 (0.96–1.20)	1.12 (0.97–1.28)		
	Size effect		0.05	0.04				0.04	0.06		
Cognitive problems/inattention	Wald’s χ^2^	10.46**			4.69*	0.64	6.22*			1.35	0.13
	OR (± CI)		1.10 (1.01–1.19)	0.99 (0.89–1.10)				1.12 (0.87–1.27)	0.99 (0.85–1.15)		
	Size effect		0.05	-0.01				0.06	-0.01		
Hyperactivity	Wald’s χ^2^	29.16***			57.40***	1.96	12.85**			36.20***	1.18
	OR (± CI)		1.03 (0.94–1.13)	1.20 (1.07–1.34)				0.99 (0.87–1.12)	1.23 (1.05–1.43)		
	Size effect		0.02	0.10				-0.01	0.11		
Oppositional symptoms	Wald’s χ^2^	20.96***			14.00***	5.38	10.49**			10.68***	3.72
	OR (± CI)		1.07 (0.96–1.20)	1.17 (1.02–1.36)				1.13 (0.95–1.33)	1.12 (0.91–1.36)		
	Size effect		0.04	0.09				0.07	0.06		

Model I included only age as a covariate, whereas Model II included several confounders: age, parental education, family income, mother’s HRSS score, gestational age and birth weight.

*AilaPreS* prenatally Aila-exposed children, *AilaPostS* postnatally Aila-exposed children, *Control* non-exposed to Aila counterparts, *OR* odds ratio, ± *CI* 95% confidence interval for odds ratio.

***p < 0.001, **p < 0.01, *p < 0.05.

**Figure 1:**
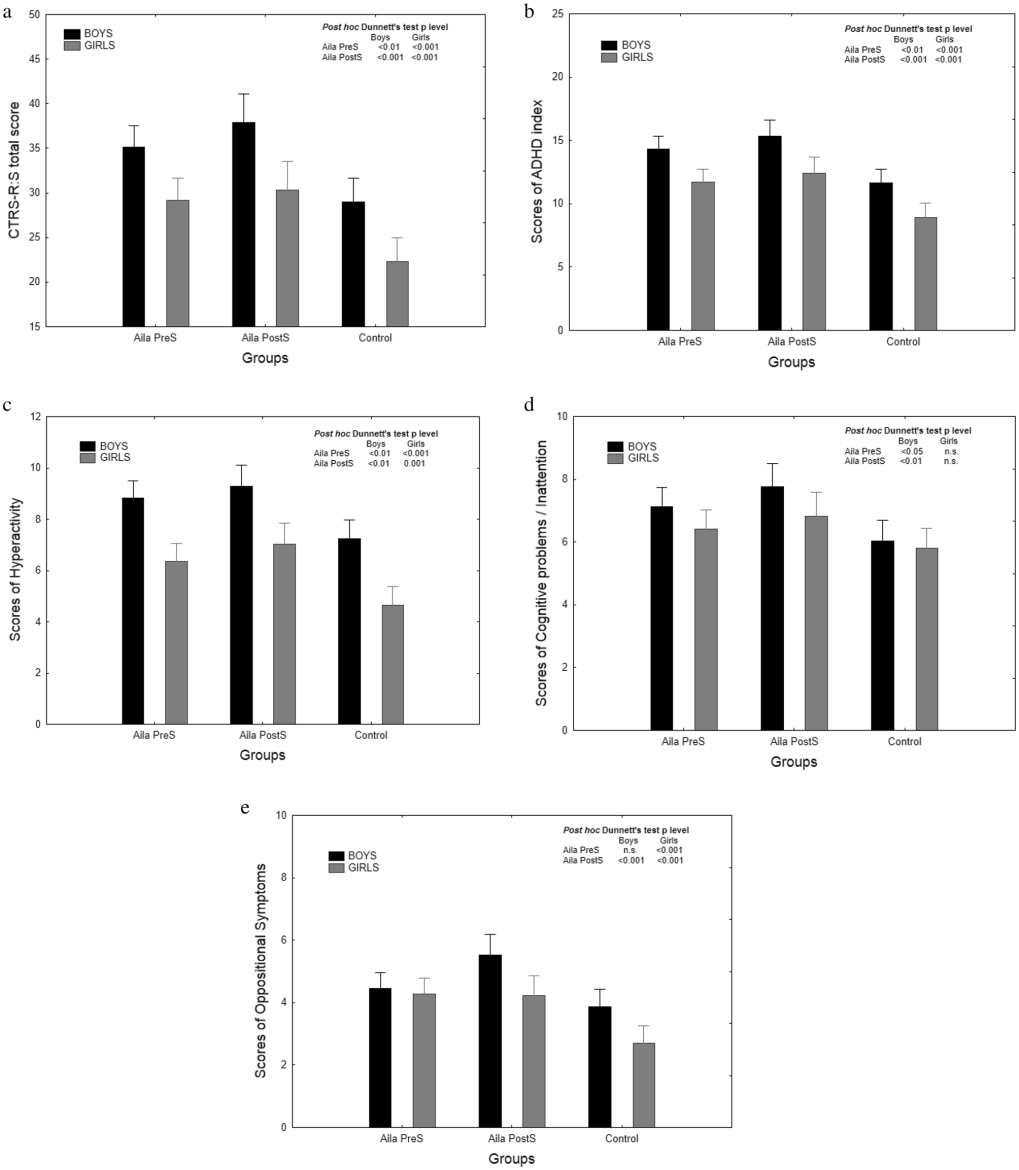
Means, CIs (confidence intervals) and Dunnett’s test results for the total scores of CTRS-R:S (**a**), ADHD Index (**b**), hyperactivity (**c**), cognitive problems/inattention (**d**) and oppositional symptoms (**e**) in boys and girls by two groups of Aila-exposed children and controls.

The highest total scores of CTRS-R:S, ADHD index and Hyperactivity were found in AilaPostS, however, the means of both exposed groups significantly differed from the control group (Fig. 1a–c). The means of Cognitive problems/inattention score were significantly different between the exposed groups and the control group in boys, but not in girls (Fig. 1d). In boys, only the AilaPostS group showed a significant difference in comparison to the control group in a mean score of Oppositional symptoms, whereas in girls both AilaPreS and AilaPostS showed significant differences in this parameter compared to the control group (Fig. 1e).

## Discussion

This study examined the effects of early exposure of children to the severe cyclonic storm Aila on the later ADHD symptoms. A few research projects, such as the 1998 Quebec Ice Storm, Canada^33,34^, the Iowa Flood Study in USA^35^ or the Queensland Flood (2011) Study in Australia^36^, utilized a natural disaster as a model for investigating the impact of prenatal stress on child development. Nevertheless, to the best of our knowledge, our is the first study that examines ADHD symptoms which are associated with exposure to a natural disaster, and with the control of the timing of exposure (pre- vs. early postnatal).

Although the moderating effect of exposure timing has been revealed in the study, the most important result we obtained was that both pre- and early postnatal exposure to severe, objective, and independent to genetic influences an adverse event was significantly associated with increased ADHD symptoms in preadolescent children compared to a control group. It is consistent with the results of previous research, conducted with diversified methodology, on the effects of prenatal^8,9^ and postnatal stress^10–12^ on ADHD symptoms. Biological mechanisms have been proposed to explain how adverse experiences affect a child’s development. Increased maternal glucocorticoids resulting from PNMS might impair intrauterine blood flow, affecting a child’s neurodevelopment leading to cognitive and behavioural consequences in later life^37^. Extensive brain growth and development occur also during the first 2 years of postnatal life. Research from both animals and humans suggest that early postnatal stress may be particularly influential for neurodevelopmental outcomes^38^.

Because parental education and income^30^ as well as the characteristics of birth and new-borns^31^ were previously found to be associated with ADHD, they could be also significant moderators of the stress-ADHD link in our study. However, the detailed analyses unravelled the fact that the main effect of exposure to a natural disaster on the summed score of CTRS-R:S (total ADHD), ADHD index, as well as Hyperactivity and Oppositional symptoms were independent of these factors. Less clear results were obtained for Cognitive problems/inattention. Furthermore, these symptoms were related to early life stress only in boys and when the analysis was not adjusted for other covariates. The *post-hoc* analysis revealed sex-specific patterns of the associations. Different effects of prenatal stress on ADHD symptoms in boys and girls were found also in other studies^39^. They might be a result of the biological differences between the sexes in the vulnerability of the dopamine transmitter system, the binding ability of the serotonin receptor 5-HT1A in the hippocampus, hypothalamic-pituitary-adrenocortical axis and the neurotrophic effect of sex hormones^39^. Some of these explanations may also be extended in the case of early postnatal stress, although confirmatory studies are warranted.

The study has limitations. ADHD is considered to be a neurodevelopmental disorder which has a primary genetic background^6^. One of the possible negative effects of ADHD is the completion of education at a lower level compared to individuals without ADHD^2^. In this study we found significant differences in the level of a mother’s education and income *per capita* between the groups exposed to cyclone Aila and the control group. This raises the question on the possible genetic source of the differences in the ADHD symptoms between the groups of examined children. Although the main effect of exposure to a natural disaster on ADHD symptoms in children remained significant in the analysis controlled for the socioeconomic status of the parents (Table 3), the between-groups differences in basic characteristics make formulating an unambiguous conclusion difficult.

In our research we have included several potentially confounding variables, nevertheless there could be a few other important factors that were not controlled. For example, we did not record factors such as a maternal social support system and differentials in coping with stress strategies which might have important moderating effects on childhood ADHD symptoms^39^. Besides, the present study did not consider other possible adverse experiences that might have occurred during later childhood development after cyclone Aila. Thus, the link between the exposure to cyclone Aila and ADHD symptoms might have emerged as well through the accumulation of the effects of other plausible Aila-dependent or independent environmental stressors in the post-Aila phase of life. For example, several cases of diarrhea were noticed due to food and water contamination following cyclone Aila rather than the storm itself. This suggests a possibility that the observed relation between the exposure to a natural disaster and ADHD symptoms may be connected to diet, microbiota and gut-brain axis. Although the latest review on the relation between microbiota and ADHD symptoms revealed no clear conclusion on their association^40^, it remains probable that diet, food contamination and pathogenic bacteria in a sensitivity window may affect brain development and cause later behavioral and cognitive symptoms. These factors were not controlled in our research.

## Conclusions

The aim of the study was to examine the association of prenatal and early postnatal exposure to a severe natural disaster with ADHD symptoms in children. The results showed that an increase in externalizing symptoms was related to exposure to the cyclonic storm, Aila. The ADHD symptoms in the Aila-exposed children might be interpreted as the effect of stress-induced modifications in the nervous system occurring as early as in the prenatal period. Nevertheless, the study suggests that the programming effect of a stressful experience is not limited to fetal life but extends at least into infancy. The effects of early stress were found to be independent of socioeconomic status and newborns’ characteristics, and are partially moderated by sex. Future research on the possible epigenetic, neurobiological and psychosocial mechanism linking early stress, particularly due to severe ecological disasters, and later behavioral symptoms is warranted.

## Data Availability

The dataset analysed during the current study is available from the corresponding author on request.
